# A Curious Case of Cutaneous Pathergy: Pyoderma Gangrenosum in the Setting of Hepatitis C and Cocaine Use

**DOI:** 10.7759/cureus.85807

**Published:** 2025-06-11

**Authors:** Yasasvhinie Santharam, Michael Cargill, Raafat Makary, Rafik Jacob

**Affiliations:** 1 Internal Medicine, University of Florida College of Medicine – Jacksonville, Jacksonville, USA; 2 Neuro-Pathology, University of Florida College of Medicine – Jacksonville, Jacksonville, USA

**Keywords:** cocaine use, hcv, hepatitis c virus, pathergy, pyoderma gangrenosum

## Abstract

Pyoderma gangrenosum (PG) is a neutrophilic dermatosis that presents with rapidly developing, painful ulcers, often associated with underlying autoimmune or hematological conditions. Pathergy, the exacerbation of skin lesions after minor trauma, is a hallmark of PG but may be present in other diseases. Hepatitis C virus (HCV) infection and cocaine use have been increasingly recognized as potential triggers of PG and pathergy. This case describes a patient with hepatitis C and active cocaine usage, without any known systemic inflammatory conditions, who developed pathergy and met the 2018 Delphi Criteria for PG.

## Introduction

Pyoderma gangrenosum (PG) is an ulcerative skin condition. While it is known that there is a neutrophil-driven reaction in PG lesions, the exact mechanism of pathophysiology behind PG remains poorly understood, likely given its rapid nature of development and association with several different underlying conditions. Typically, PG has been associated with autoimmune disorders such as inflammatory bowel disease (IBD), or hematological disorders such as myelodysplastic syndrome, monoclonal gammopathy of undetermined significance, or acute myeloid leukemia [1,2]. Though a lesser-established association, PG has also been associated with hepatitis, particularly HCV. In one case series of 103 patients with PG at two Boston hospitals, for instance, 9% were found to have concomitant hepatitis [3]. Several case reports have documented the hepatitis-PG association over the past few decades, as well as instances of hepatitis C virus (HCV)-related vasculitis/cryoglobulinemic vasculitis associated with PG [4-6]. In addition to hepatitis C, the use of cocaine has increasingly been recognized as a trigger for PG, especially when the drug has been adulterated with levamisole in the manufacturing process, causing levamisole-induced vasculitis [7].

## Case presentation

**Figure 1:**
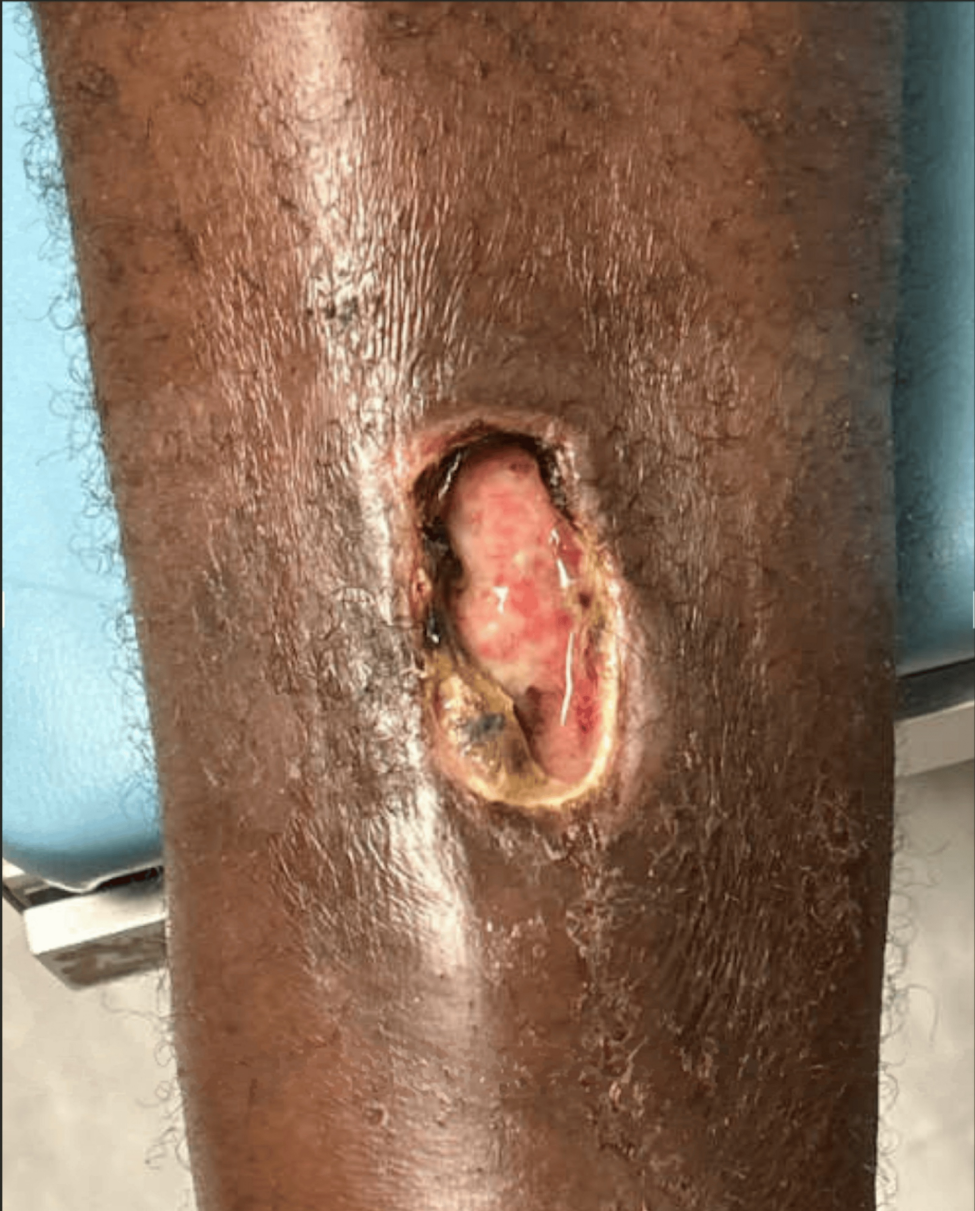
Nonpurulent lesion on the anterior shin with surrounding granulation tissue and violaceous borders on the initial day of presentation

A 64-year-old male with a history of chronic untreated hepatitis C (viral load of 1.5 million IU/mL when tested 4 years prior and 4 months prior to this visit), chronic active weekly cocaine use, and 30 pack/year ongoing tobacco use presented to the urgent care clinic with a 2-week history of a painful, growing ulceration on his right anterior shin. The ulcer had begun as a bruise after the patient had sustained a mechanical injury to his shin while moving a large pile of wood. The patient had gone to the emergency department on the day of the injury, where he was noted to have a painful bruise. On the third day, the patient awoke to find that the bruise had developed into an open lesion, despite no further mechanical injury or manipulation of the site. The wound remained unchanged despite the patient’s use of over-the-counter antimicrobial gel, cleansing with water, and application of bandages. The patient presented to an outpatient clinic on day 14 with a physical examination significant for a tender, nonpurulent lesion (Figure 1) with inlaid pink granulation surrounded by necrotic tissue and violaceous borders, characteristic of PG.

**Figure 2:**
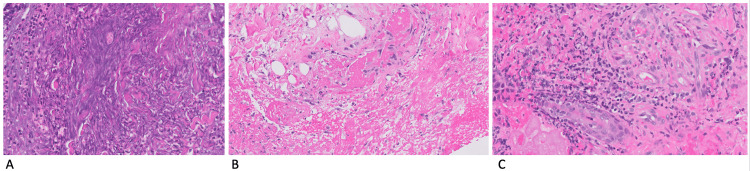
Pathology images from a skin biopsy of the edge of the ulcer A: Gangrenous necrotic exudate with neutrophils (H&E stain ×20), B: fibrin and fibrin thrombi in small blood vessels (H&E stain ×20), C: folliculitis and neutrophils in capillary walls (H&E stain ×20).

History and testing were negative for coagulation disorders, malignancy, infection, or IBD. A biopsy of the wound showed gangrenous tissue necrosis with acute inflammation (Figure 2A), fibrin and fibrin thrombi in small blood vessels (Figure 2B), and focal neutrophilic folliculitis in the walls of dermal vessels (Figure 2C). There was no active infection in the wound.

**Figure 3:**
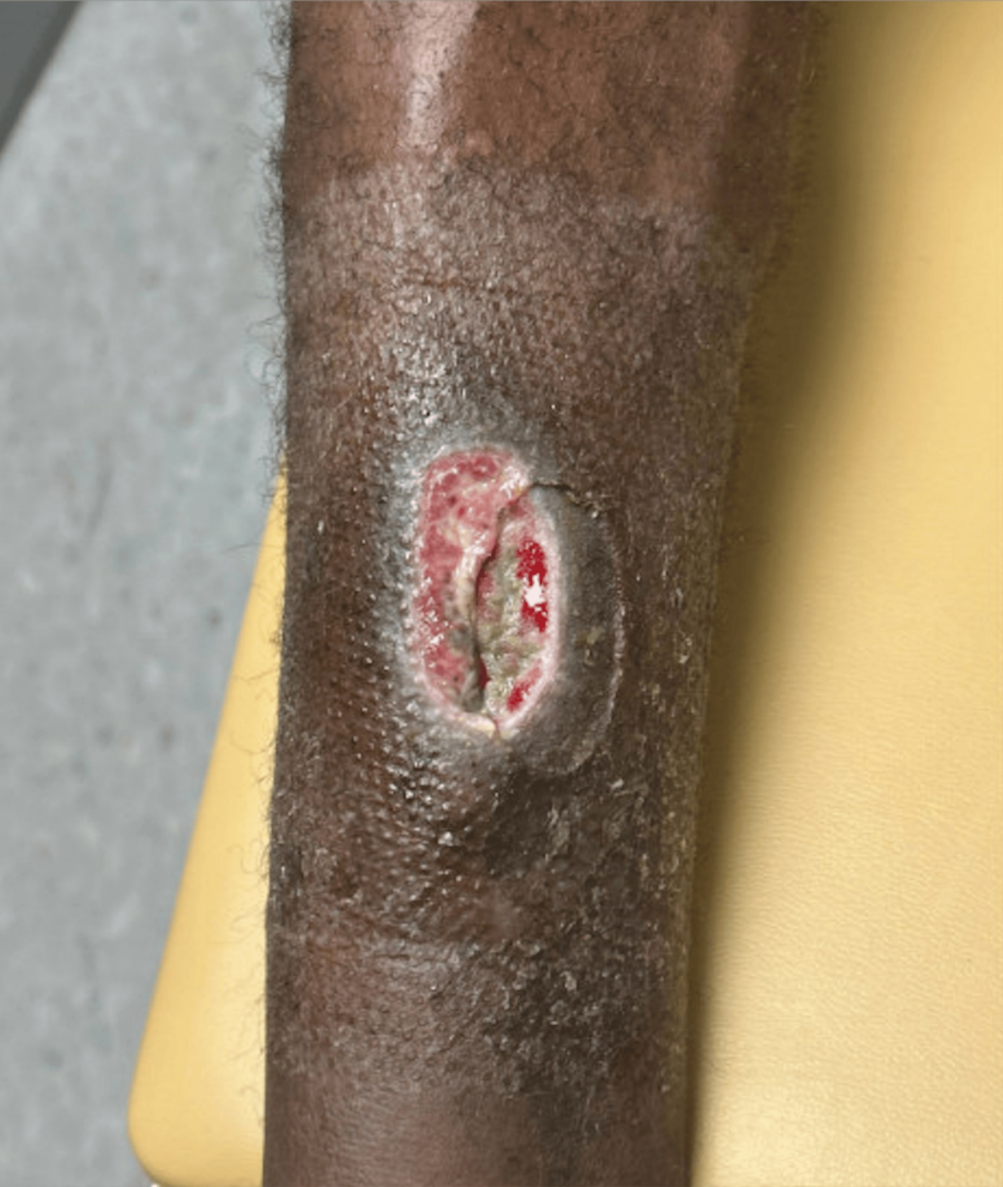
Well-healing lesion on the anterior shin filled with granulation tissue two weeks after initial presentation, after twice-weekly wound care and daily treatment with betamethasone ointment

After twice-weekly wound care and two weeks of treatment with betamethasone ointment, the lesions had healed significantly (Figure 3); however, the patient ultimately lost to follow-up.

## Discussion

PG has been associated with several conditions such as IBD, rheumatoid arthritis, seronegative spondyloarthropathies, solid and hematologic malignancies, myelodysplastic syndrome, hepatitis C, and vasculitides [1,2,5,6,8,9]. Although positive for HCV and admitting to frequently doing cocaine, our patient lacked symptoms such as fatigue, joint pain, or Raynaud’s, which would have indicated systemic vasculitis. As the patient was lost to follow-up, he did not complete the anti-neutrophil cytoplasmic antibody (ANCA) test for vasculitides. However, within the workup he was able to complete, he was negative for antinuclear antibodies (ANA), negative for any hematologic disorders, and did not have any symptoms of IBD or other underlying malignancies.

With preceding trauma and speed of the wound development, as well as the lack of edema, venous stasis, or abnormal pulses in the lower extremities, it is unlikely that the ulcer was due to venous or arterial insufficiency. Local infection was excluded, as the patient did not have fevers, chills, or tachycardia, had no cellulitis or local purulent infection at the site of the wound, and had marked healing with steroid cream. Another consideration was a medication-induced etiology, as some prescription drugs, including isotretinoin, propylthiouracil, and sunitinib, have been associated with PG [7,8]. As he was not taking any long-term medications, a medication-induced PG was also excluded. Although less common, our case highlights one of the instances where pathergy can occur in the setting of HCV and cocaine use despite no accompanying symptoms of underlying systemic vasculitis or other disorders, and a diagnosis of PG was met using the criteria below.

While PG has historically been considered a diagnosis of exclusion, recent efforts have been made to develop diagnostic frameworks for this condition. One major framework, the 2018 Delphi Criteria, includes one major criterion as well as eight minor criteria, as outlined in Table 1. The paper that outlined these criteria ran an analysis that determined having one major criterion as well as at least four out of eight of the minor criteria maximized discrimination and yielded a sensitivity and specificity of 86% and 90%, respectively, for the diagnosis of PG [10].

**Table 1:** 2018 Delphi Criteria for Diagnosis of Pyoderma Gangrenosum

Major Criterion	A biopsy of the ulcer edge must demonstrate a neutrophilic infiltrate.
Minor Criteria	Exclusion of infection
	Pathergy
	History of IBD or inflammatory arthritis
	History of papule, pustule, or vesicle ulcerating within four days of appearing
	Peripheral erythema, undermining border, and tenderness of the ulceration site
	Multiple ulcerations, at least one on the anterior lower leg
	Cribriform scars at healed ulcer sites
	Decreased ulcer size within one month of initiating immunosuppressive medications

Given the clinical presentation along with the known history of hepatitis C and active cocaine use, the violaceous erythema on the borders of the lesion, and the surgical pathology findings of neutrophilic infiltration, a diagnosis of PG was made, supported by fulfillment of the major criterion and five of the minor criterion of the Delphi criteria.

Although there is no universally agreed-upon set of guidelines for the management of PG, the treatment of PG generally involves some form of immunosuppressive medication, using clinical judgment. If there are large lesions (greater than 4cm in length), multiple lesions, involvement of deeper structures such as muscles within the ulcerated regions, or recurrent ulcerations, then oral or IV therapy may be warranted to gain faster control to decrease the inflammation [11]. If the ulcers do not meet the aforementioned criteria, then the first-line therapy tends to be topical or local intra-lesionally injected steroids, which have level 2B evidence per a recent literature review in the American Journal of Dermatology [12]. In this case, our patient underwent treatment of his PG ulcer with betamethasone ointment, as he had only one ulcer that did not involve deeper structures, and he voiced a preference and higher likelihood of adherence with ointments over taking oral medications. As the patient was adherent with this therapy and presented with notable improvement in healing after two weeks, he was asked to continue with the topical steroid and did not present again with further issues, responding to a phone call a month later stating that his ulcerations had fully healed. As any patient with a documented instance of PG should be advised, this patient was told to take caution to avoid further trauma to the skin if possible, to decrease the likelihood of future events.

## Conclusions

PG can arise in individuals with untreated hepatitis C and cocaine use in the absence of underlying autoimmune or hematologic disorders. Diagnostic frameworks, such as the 2018 Delphi criteria, are clinically relevant tools in the diagnosis of PG in patients with complex comorbidities.
